# Supplementary material for: Transcriptional Profiling Reveals Adaptive Response and Tolerance to Lactic Acid Stress in Pichia kudriavzevii
Source: Foods. 2022 Sep 6;11(18):2725. doi: 10.3390/foods11182725 (PMC9498142; doi:10.3390/foods11182725)
Supplement: Supplementary file 1 [file foods-11-02725-s001.zip › foods-1870636-supplementary.pdf]

Supplementary Materials

Transcriptional Profiling Reveals Adaptive Response and Tolerance to Lactic Acid Stress in *Pichia kudriavzevii*

Hai Du †, Yan Fu †, Nan Deng and Yan Xu *

Laboratory of Brewing Microbiology and Applied Enzymology, Key Laboratory of Industrial Biotechnology of Ministry of Education, School of Biotechnology, Jiangnan University, 1800 Lihu Avenue, Wuxi 214122, China

* Correspondence: yxu@jiangnan.edu.cn; Tel.: +86-510-85964112

† These authors contributed equally to this work.

Supplemental Tables

Table S1. Fold change in growth and metabolism of *P. kudriavzevii* C-16 and its type strain ATCC 24210 under different concentrations of lactic acid stress.

Concentration of lactic acid （g L^{-1}）	OD_{600} of the end of fermentation	Specific growth rate	Consumption of lactic acid	Consumption rate of lactic acid（12 h）	Consumption rate of lactic acid（24 h）
20	1.14	1.06	1.37	1.24	2.36
30	1.20	1.09	2.83	2.70	2.55
40	1.16	1.05	2.04	1.57	2.93

Note: Fold change is the ratio of *P. kudriavzevii* C-16 to the type strain ATCC 24210.

Table S2. Genome comparison between reads and reference sequence.

Sample	clean reads (million)	Total mapped reads (%)	Multiple mapped reads (%)	Uniquely mapped reads (%)
P. kudriavzevii C-16 cultured for 12 h	48.27	97.06	1.82	95.24
P. kudriavzevii C-16 cultured for 24 h	56.87	97.19	2.27	94.93
P. kudriavzevii ATCC 24210 cultured for 12 h	49.86	96.89	2.25	94.64
P. kudriavzevii ATCC 24210 cultured for 24 h	46.29	96.85	1.95	94.90

Table S3. Differently expressed genes related to pyruvate metabolism.

Gene	Function	Fold change （12 h）	Fold change （24 h）
lldD	L-lactate dehydrogenase (cytochrome)	6.13	11.02
LEU4	2-Isopropylmalate synthase	2.60	3.01
MAE1	Malate dehydrogenase (oxaloacetate-decarboxylating)	2.33	1.08
PCK1	Phosphoenolpyruvate carboxykinase (ATP)	2.64	1.60
ACS1	Acetyl-CoA synthetase	1.32	1.89

Note: Fold change is the ratio of *P. kudriavzevii* C-16 to the type strain ATCC 24210.

Table S4. Fold change of transcription of the gene *SNQ2*.

Gene ID	Function	Fold change （12 h）	Fold change （24 h）
C5L36_0C11740		31.13	54.80
C5L36_0B06280	ATP-binding cassette transporter *SNQ2*	3.95	4.09
C5L36_0B12900		1.27	1.90

Note: Fold change is the ratio of *P. kudriavzevii* C-16 to the type strain ATCC 24210.

Table S5. Differently expressed genes related to glutamate metabolic.

Gene	Function	Fold change（12 h）	Fold change（24 h）
GAD1	glutamate decarboxylase	2.45	1.74
UGA2	succinate-semialdehyde dehydrogenase	2.23	1.75

Note: Fold change is the ratio of *P. kudriavzevii* C-16 to the type strain ATCC 24210.

Table S6. Differently expressed genes related to biosynthesis of amino acids.

Gene	Function	Fold change（12 h）	Fold change（24 h）
LEU2	3-isopropylmalate dehydrogenase	2.09	1.81
ILV5	ketol-acid reductoisomerase	2.66	1.97
LEU4	2-isopropylmalate synthase	2.60	3.01
LEU1	3-isopropylmalate dehydratase	4.49	4.43

Note: Fold change is the ratio of *P. kudriavzevii* C-16 to the type strain ATCC 24210.

Supplemental Figures

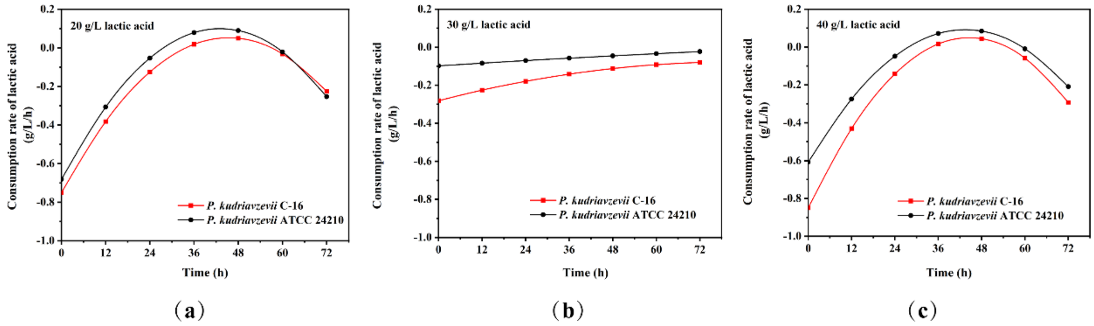

Figure S1. Lactic acid consumption rate of *P. kudriavzevii* C-16 and the type strain ATCC 24210 under different concentrations of lactic acid stress. (a) Under 20 g/L lactic acid stress; (b) under 30 g/L lactic acid stress; (c) under 40 g/L lactic acid stress. The initial pH of the culture medium was adjusted to 3.5.

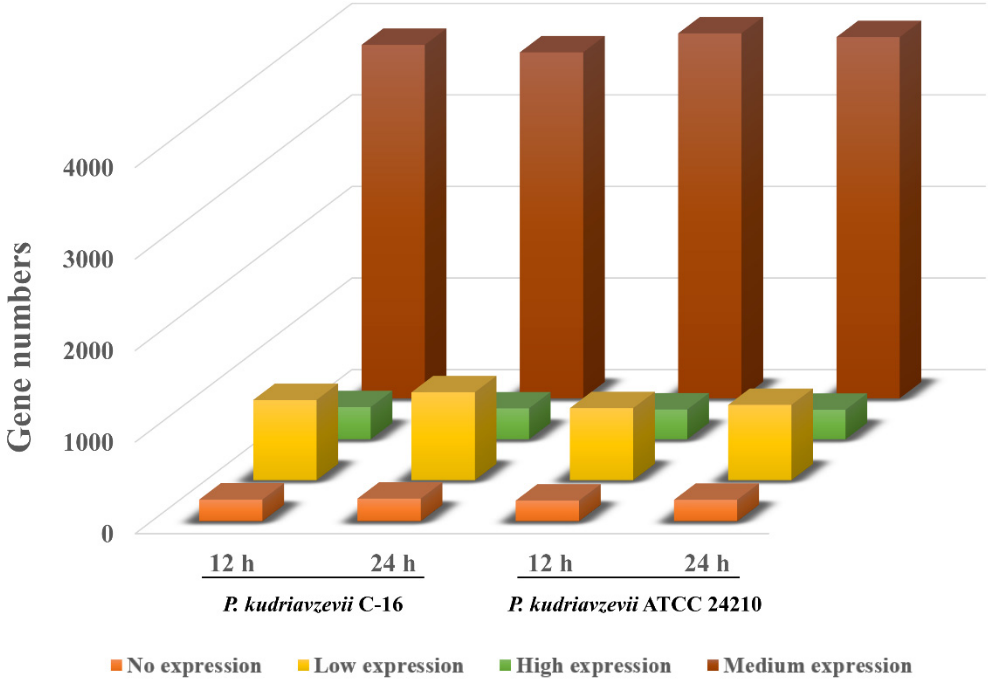

Figure S2. The abundance of the gene expression of *P. kudriavzevii* C-16 and its type strain ATCC 24210 throughout the cultivation period. The transcripts were assessed based on FPKM values: high expression (FPKM ≥ 500), medium expression (15 ≤ FPKM < 500), low expression (1 ≤ FPKM < 15), and no expression (0 ≤ FPKM < 1).
